# A national atlas of tsetse and African animal trypanosomosis in Mali

**DOI:** 10.1186/s13071-019-3721-3

**Published:** 2019-10-09

**Authors:** Boucader Diarra, Modibo Diarra, Oumar Diall, Boubacar Bass, Youssouf Sanogo, Etienne Coulibaly, Mahamadou Sylla, Weining Zhao, Massimo Paone, Giuliano Cecchi

**Affiliations:** 1 Direction Nationale des Services Vétérinaires, Cellule de Coordination de la Lutte contre les Mouches tsé-tsé et les Trypanosomoses animales (CCLMT), Bamako, Mali; 2 Ministère de l’Agriculture, Comité National de la Recherche Agronomique (CNRA), Bamako, Mali; 3 Ministère de l’Elevage et de la Pêche, Bamako, Mali; 4 Food and Agriculture Organization of the United Nations (FAO), Animal Production and Health Division, Rome, Italy

**Keywords:** Mali, Tsetse, African animal trypanosomosis, GIS, Atlas, Database, Epidemiology

## Abstract

**Background:**

Tsetse-transmitted trypanosomosis is a deadly, neglected tropical disease and a major challenge for mixed crop-livestock agriculture in sub-Saharan Africa. It is caused by several species of the genus *Trypanosoma*. Information on the occurrence of tsetse flies and African animal trypanosomosis (AAT) is available for different areas of Mali. However, these data have never been harmonized and centralized, which prevents the development of comprehensive epidemiological maps and constrains an evidence-based planning of control actions. To address this challenge, we created a dynamic geo-spatial database of tsetse and AAT distribution in Mali.

**Methods:**

A digital repository containing epidemiological data collected between 2000 and 2018 was assembled. In addition to scientific publications, the repository includes field datasheets, technical reports and other grey literature. The data were verified, harmonized, georeferenced and integrated into a single spatially-explicit database.

**Results:**

For the tsetse component, approximately 19,000 trapping records, corresponding to 6000 distinct trapping locations and 38,000 flies were included in the database. *Glossina palpalis gambiensis* was the most widespread and abundant species, and it was found in the southern, southern-central and western parts of the country. *Glossina tachinoides* was only found in the South. Only a few specimens of *Glossina morsitans submorsitans* were detected. For the AAT component, approximately 1000 survey records were included, corresponding to 450 distinct survey sites and 37,000 tested bovines. AAT was found in all surveyed regions, although data for the tsetse-free North and North-East are lacking. *Trypanosoma vivax* and *Trypanosoma congolense* were the dominant species, while *Trypanosoma brucei* infections were much less numerous.

**Conclusions:**

The atlas of tsetse and AAT in Mali provides a synoptic view of the vector and disease situation at the national level. Still, major geographical gaps affect the North, the North-East and the West, and there is also a severe lack of data over the past five years. Trypanosomosis remains a major animal health problem in Mali. However, despite its prevalence and distribution, monitoring and control activities are presently very limited. Efforts should be made to strengthen the progressive control of AAT in Mali, and the atlas provides a new tool to identify priority areas for intervention.
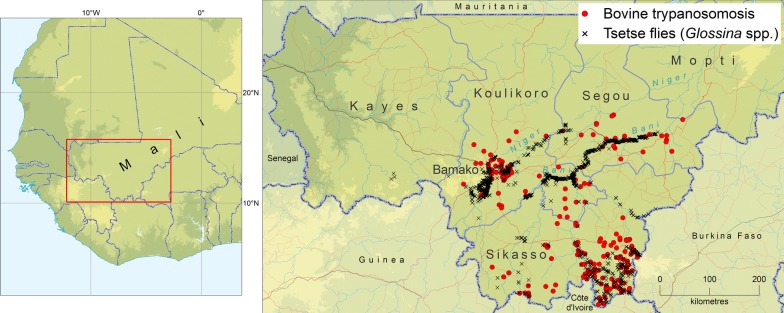

## Background

Tsetse-transmitted animal trypanosomosis (also called ‘nagana’) constrains agricultural production in more than 10 million km^2^ in sub-Saharan Africa, some of which hold the continent’s greatest potential for expanded agricultural production [1]. It has been argued that, historically, trypanosomosis reduced the ability of African populations to generate an agricultural surplus and that the current economic performance of the continent is still heavily affected by tsetse today [2].

African animal trypanosomosis (AAT) is a wasting disease caused by unicellular protozoan parasites of the genus *Trypanosoma* (order Kinetoplastida) [3]. It affects ruminants, camels, equines, swine and carnivores and its main pathogenic agents are *T. vivax*, *T. congolense*, *T. brucei* and *T. simiae* [4]. AAT is cyclically transmitted by the bite of infected tsetse flies (*Glossina* spp.), but the infection, especially with *T. vivax,* can also be mechanically transmitted by other biting flies (most notably *Tabanus* and *Stomoxys* spp.) [5]. Two subspecies of *T. brucei*, i.e. *T. b. rhodesiense* and *T. b. gambiense*, are responsible for the human form of trypanosomosis, also called sleeping sickness [6], while *T. b. brucei* is not pathogenic to humans.

Mali is a predominantly agro-pastoral country. However, it is unable to meet its needs for animal products, including meat, milk and dairy products, because of very low levels of productivity. At the country level, up to 3 million bovines are estimated to be at risk of trypanosomosis [7, 8], which is the main vector-borne disease in the country. In particular, trypanosomosis is widespread in those regions having the highest agricultural potential (e.g. for cotton production). The latest estimates place tsetse infestation at 240,000 km^2^ [9]. With regard to human African trypanosomosis (HAT), there has not been a recorded case for over twenty years in Mali [10], the latest case having been detected in 1995 [11].

In the present paper we refer to five geographical zones in Mali, i.e. southern (Sikasso region), southern-central (south of the Ségou and Koulikoro regions, the latter including the peri-urban area of the capital Bamako), western (southern part of the Kayes region), northern (northern parts of the Kayes, Koulikoro and Ségou regions) and northern-eastern (Mopti, Tombouctou, Gao and Kidal regions).

In Mali, tsetse flies are only found in the southern, southern-central and western zones. The latest review on the tsetse distribution in Mali, published over 20 years ago [7], reported the presence of four species. The most abundant ones both belonged to the riverine group, i.e. *Glossina palpalis gambiensis* and *G. tachinoides*, while *G. morsitans submorsitans* and *G. longipalpis* (two species of the savannah group) were reported to be far less common. As regards tsetse-transmitted animal-infective trypanosomes, *T. congolense*, *T. vivax* and *T. b. brucei* are the three main species recorded in Mali.

The control of tsetse and trypanosomosis is considered by the Malian authorities as an important strategic component to increase agricultural production, ensure food security, and improve human and animal health. In fact, since the 1970s, a number of institutions, projects and programmes, some of which research-oriented, have focused on trypanosomosis control. These include the Central Veterinary Laboratory (Laboratoire Central Vétérinaire, LCV), the Central Unit for Tsetse and Trypanosomosis Control (Unité Centrale de Lutte contre les tsé-tsé et les Trypanosomoses, UCLT), and the Tsetse Fly and Animal Trypanosomosis Control Project in Mali (Projet de Lutte contre les Mouches tsé-tsé et les Trypanosomoses animales au Mali, PLMT). Mali also joined the Pan-African Tsetse and Trypanosomosis Eradication Campaign (PATTEC), an initiative of the African Union endorsed by the African Heads of State and Government [12].

In 2015, the Coordination Unit for Tsetse and Animal Trypanosomosis Control (Cellule de Coordination de la Lutte contre les Mouches tsé-tsé et les Trypanosomoses animales, CCLMT) was established and tasked with coordinating interventions against tsetse and trypanosomosis at the national level. The CCLMT is supported by the LCV, especially in matters related to operational research.

The CCLMT recently developed a new national strategy in line with the Progressive Control Pathway (PCP) for AAT [13]. PCPs are risk-based, stepwise approaches initially developed for foot and mouth disease [14], and subsequently adapted to a number of other diseases [13, 15–17]. One of the basic requirements to advance along the PCP is the mapping of AAT risk for evidence-based decision making. This requires good, spatially explicit knowledge of AAT and tsetse occurrence. However, as it is the case in most affected countries, no national map of AAT distribution and endemicity is available in Mali, and the most widely used maps of tsetse distribution date back to the 1970’s [18, 19], with only sporadic updates in the following two decades [7]. The lack of synoptic information on the disease and its main vectors limits the ability to plan control actions in a rational manner, and to estimate the impact of interventions. To address this challenge, in 2015 the CCLMT launched the development of a dynamic geo-spatial database of AAT and tsetse distribution in Mali (i.e. the Atlas). The initiative is backed by the commitment of the Malian government and it is technically supported by FAO within framework of the Programme against African trypanosomosis (PAAT) [20, 21]. The development of this tool was accompanied by capacity building in data management and geographical information systems (GIS).

## Methods

The Atlas of tsetse and AAT in Mali broadly follows the methodology developed by FAO for the continental Atlas [22, 23], which was already adapted at the national level in Sudan [24]. However, while the FAO continental atlas is only based on peer-reviewed scientific publications, national atlases aim to include all epidemiological data collected in the country, be it published or unpublished.

The atlas of tsetse and AAT in Mali includes data collected in the period 2000–2018. The majority of data were provided by national institutions that are or have been involved in tsetse and trypanosomosis research and control (i.e. UCLT, LCV, PLMT, and CCLMT, the latter also playing a coordinating role in the initiative).

### Data sources

The data used as input for the atlas were normally collected in the context of AAT control activities, and they include both baseline (i.e. pre-intervention) and monitoring surveys (i.e. during or post-intervention). Data collected in these settings are rarely published. An additional source of data is provided by research activities, whose outputs are generally disseminated through scientific publications. The full list of scientific papers whose results have been included in the atlas is provided in Additional file 1: Text S1. For these publications, the unpublished raw data were obtained from authors and used for the national atlas (unlike the FAO continental atlas, which only relies on the information and data that can be extracted from the publications themselves).

From the geographical point of view, the southern and the southern-central part of the country have been the main targets of control and monitoring activities in the last two decades, and therefore they provided a substantial amount of data for the atlas. Another major source of data was a project carried out between 2006 and 2013 in the framework of the PATTEC initiative and funded by the African Development Bank (AfDB). The project targeted the South-Centre, and in particular the Bani river basin and part of the Niger basin (with a focus on the peri-urban areas around Bamako).

#### Tsetse flies

Entomological data on tsetse distribution and abundance are usually collected to assess the impact of control interventions. In Mali, the insecticide-treated cattle (ITC) and insecticide-treated targets/traps (ITT) are the most common vector control techniques. Baseline studies measure the vectors’ occurrence before interventions while monitoring studies evaluate the impact during and after the operations.

In the field, entomological data are usually recorded by means of standardized paper forms (recording sheets) [25]. Among other elements, these forms include such information items as the name of the surveyed location, its coordinates and administrative units (which in Mali are called régions, cercles and communes), the date of the survey, the time and duration of trap deployment and removal, and the number, species and sex of trapped tsetse flies. On average, a single recording sheet includes results from approximately 55 traps.

These data are complemented by information on the tsetse control activities carried out in the area, if any. This type of information is normally available from narrative mission reports rather than from the data recording sheets.

#### African animal trypanosomosis

As for the case of tsetse data, AAT data are mainly collected during baseline or monitoring epidemiological investigations. In these contexts, tested animals are normally selected randomly, and, to a large extent, the survey sites are also selected randomly. By contrast, in a few studies that looked at trypanocidal drug resistance, high-risk villages are purposely selected from those villages having the highest disease prevalence and risk [26, 27].

In terms of data recording, the standard parasitological sheets include the data sources (including the institution in charge), the name, geographical coordinates and administrative units pertaining to the survey village or site, the date of the survey and the sample size (i.e. the number of animals tested). The sheets also include the number of AAT-positive animals by trypanosome species, and the haematocrit (packed cell volume, PCV). A single AAT recording sheet typically includes information on between 50 and 100 animals. The possible use of trypanocidal drugs (including the type of drug) and the possible application of epicutaneous treatments of cattle for tsetse control (i.e. ITC) are also recorded.

### Structure of the atlas

The atlas of tsetse and AAT in Mali is composed of two major elements: the data repository and the database.

#### Data repository

The data repository includes digital copies of all input files used to build the database. It stores spreadsheets, scientific articles (as listed in Additional file 1: Text S1), reports, briefs, theses, and further grey literature. At a first level, the tsetse and AAT are distinguished, and they are subsequently structured into subfolders related to different contributing institutions. File names include the time and area of the surveys to facilitate consultation. Most field data recording sheets are stored in the repository as digital spreadsheets, because scanned copies of the original hard copies are rarely available.

#### The database: tsetse component

In the database, all entomological data are recorded in one single table (see Additional file 2: Text S2). For each record, the table includes the data source, the location (e.g. village) and related administrative units, the geographical coordinates of the trapping site (latitude and longitude in decimal degrees on WGS84 datum), the survey period, the type of trap, the attractant used (if any) and the duration of trapping. The results of the survey are recorded in terms of tsetse species, number of flies caught, apparent density (i.e. flies/trap/day) and sex. Information on the possible presence of tsetse control activities in the surveyed area is also recorded.

The biconical trap [28] without attractants was the trap used in all surveys. Furthermore, in surveyed areas, traps were normally deployed in the most favourable habitat for tsetse, in particular in the riverine vegetation. Traps were geo-referenced with GPS and the duration of trapping was normally 24 hours. In rare occasions, traps were deployed for 48 or 72 hours.

#### The database: African animal trypanosomosis component

While the tsetse component of the database only includes one table, data for the AAT component are split into three different tables: data sources, geographical data, and epidemiological data. This three-table structure follows more closely the one developed for the continental Atlas [23].

The table on data sources summarizes information on the input files stored in the repository. Each source is given a unique identifier and the following information is recorded: the author and institution that generated the source, its title, and the year of production.

The table on geographical data includes information on the survey sites, i.e. location name and its geographical coordinates and administrative units. A unique numerical identifier is given to each site, which is used to link the geographical data to the epidemiological data. All survey sites are represented as point entities in the resulting maps.

The table on epidemiological data summarizes the results of the epidemiological investigations. In this table, each record includes: survey period, diagnostic method, sample size (i.e. number of animals tested), animal species, animal breed, age range, sex, husbandry system and haematocrit/PCV at the herd level. Trypanosomal infections are captured in terms of presence/absence, number of infected animals and prevalence rate. The species of trypanosomes (i.e. *T. vivax*, *T. congolense* and *T. brucei*) are also recorded, as well as mixed infections with more than one species. Information on recent or ongoing interventions against tsetse and on the possible use of trypanocidal drugs (if any) is also recorded. The type of animal sampling (random or purposeful) is recorded, if the information is available. The unique identifiers enable the epidemiological records to be linked to the corresponding sources and geographical entities (see Additional file 2: Text S2).

All parasitological data included in the atlas of Mali concern a single livestock species, i.e. cattle, and all surveys relied on the same diagnostic method, i.e. the buffy coat technique (BCT), [29].

### Atlas development process

Various steps were needed to develop the atlas. First, all available input data were collated from the different institutions. If only available in hard copy, the data were digitized (i.e. entered into digital spreadsheets). All digital files were subsequently assembled in the data repository.

The merging of the different datasets into a single database required a systematic process of standardization, harmonization and verification. For example, the format of geographical coordinates were standardized as latitude and longitude (decimal degrees on WGS84 datum); if projected coordinates [i.e. Universal Transverse Mercator (UTM)] were reported in the input files, they were converted into latitude and longitude. Harmonization was also needed to record information on breeds, husbandry systems, names of geographical locations and the related administrative units.

A sizeable number of AAT epidemiological records initially lacked GPS-based geographical coordinates, even though they normally included the name of the survey site. In these cases, coordinates were extracted from alternate sources, e.g. gazetteers (i.e. geographical indexes containing the names and coordinates of a range of geographical locations), and in particular the Geonet Names Server developed by the Unites States National Geospatial-Intelligence Agency (NGA) [30].

Also, input files often lacked information on whether interventions against tsetse were taking place or trypanocidal drugs were being used in the study areas at the time of surveys. This is because standard data recording sheets do not contemplate a column to record this type of information. As a consequence, further efforts were needed in an attempt partly to fill these information gaps and to record the related information into the database.

## Results

**Fig. 1:**
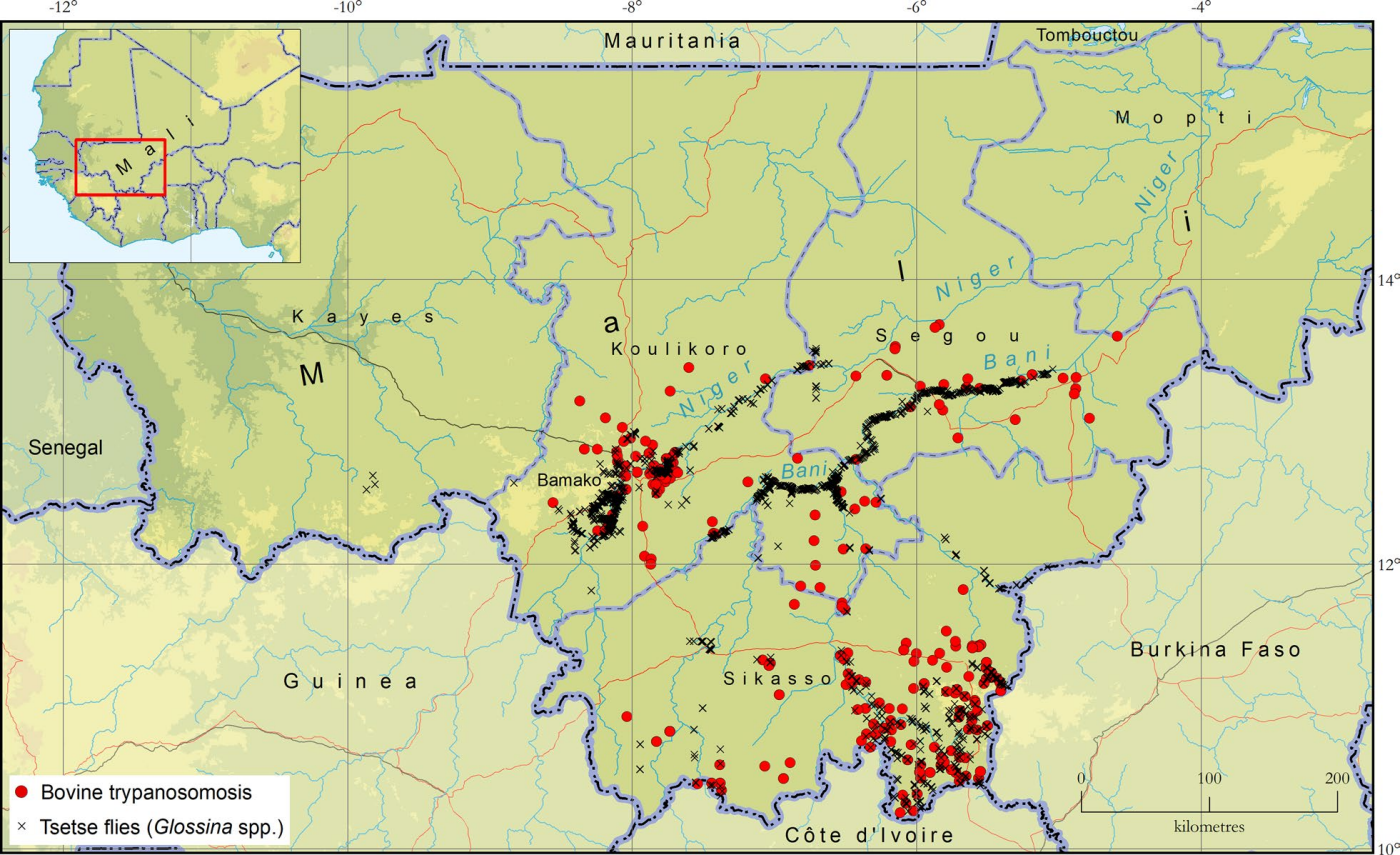
The occurrence of African animal trypanosomosis and tsetse flies (Genus *Glossina*) in Mali. Data collection period: 2000–2018

Approximately 1024 data recording sheets were included in the atlas repository (i.e. 305 for the tsetse and 719 for the AAT component, respectively). The review of the published and grey literature also enabled to identify 23 eligible documents. These documents include fourteen articles published in scientific journals (Additional file 1: Text S1), eight additional documents including five PhD theses, one MSc thesis and three BSc theses. The results of tsetse and AAT mapping are summarized in Fig. 1.

### Tsetse distribution

Regarding the tsetse component of the database, approximately 6000 trapping sites were recorded, corresponding to 19,000 entomological records. Geographical coordinates could be identified for approximately 90% of the trapping sites. A total of approximately 38,000 tsetse fly catches were included in the database.

These entomological surveys have mainly concerned the South-Centre, and, at a lower intensity, the South (Sikasso region). Data are very limited in the West (Kayes region) and completely lacking from the North and the North-East.

**Fig. 2:**
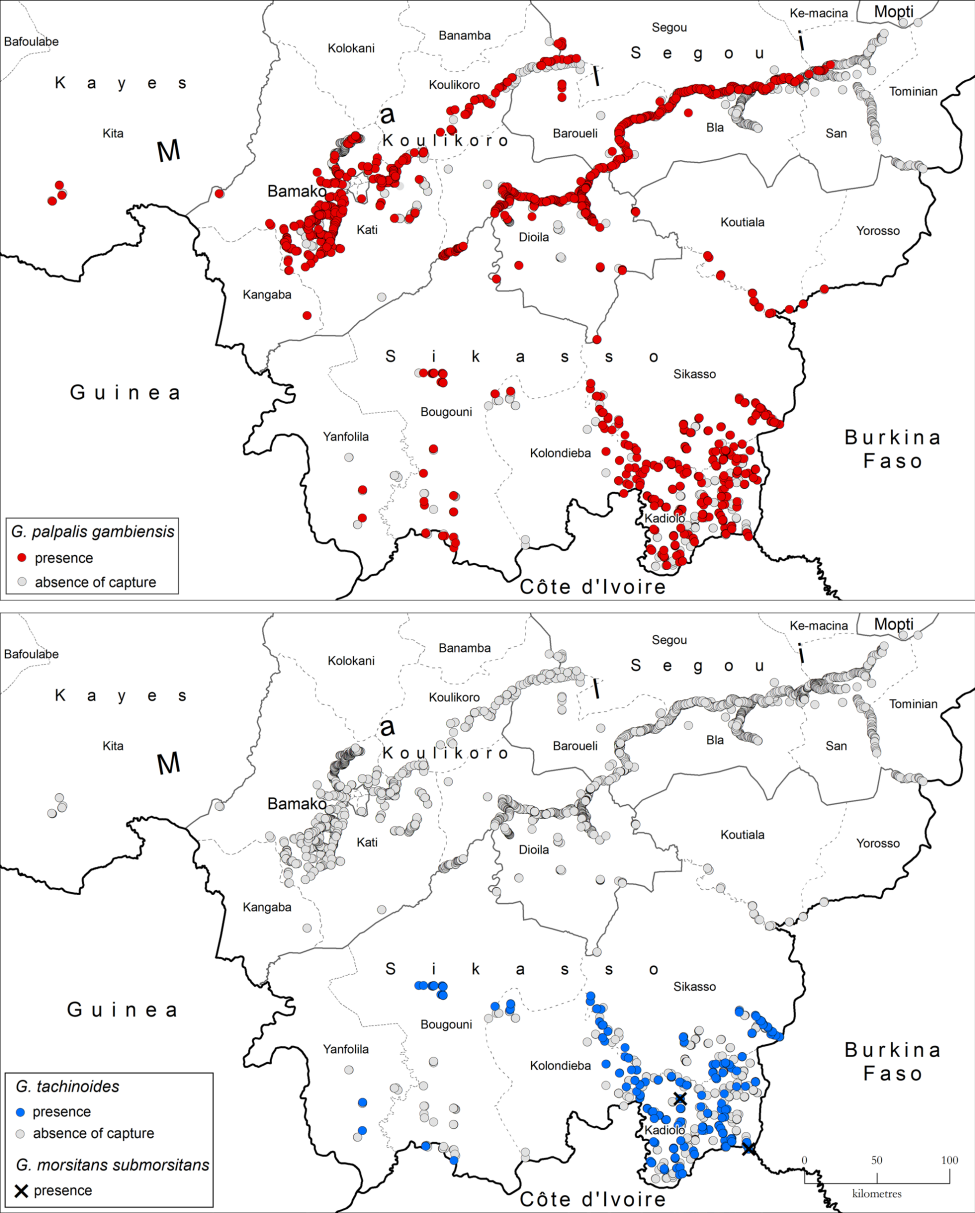
Presence and absence of capture of *G. palpalis gambiensis*, *G. tachinoides* and *G. morsitans submorsitans* in Mali. Data collection period: 2000–2018. Trapping device: biconical trap [28] without attractants

Tsetse flies were found virtually in all surveyed areas (Fig. 2), with the exception of the northern-eastern part of the Bani river basin. The vast majority of the tsetse flies recorded in the atlas (i.e. 98%) belong to the species *G. palpalis gambiensis*, the remaining 2% belonging to *G. tachinoides*. Only four *G. morsitans submorsitans* were found. From the geographical standpoint, *G. p. gambiensis* was found to be widespread across the surveyed areas, while *G. tachinoides* occurrence was limited to southern part of the country (Sikasso region).

### African animal trypanosomosis distribution

The trypanosomal infection status of 36,728 tested cattle was recorded in the AAT component of the database, corresponding to 465 distinct survey sites.

AAT was found in all the tsetse-infested surveyed regions, although limited information is available in the West (Kayes region). No information at all is available for the vast tsetse-free areas in the northern and northern-eastern part of the country.

**Fig. 3:**
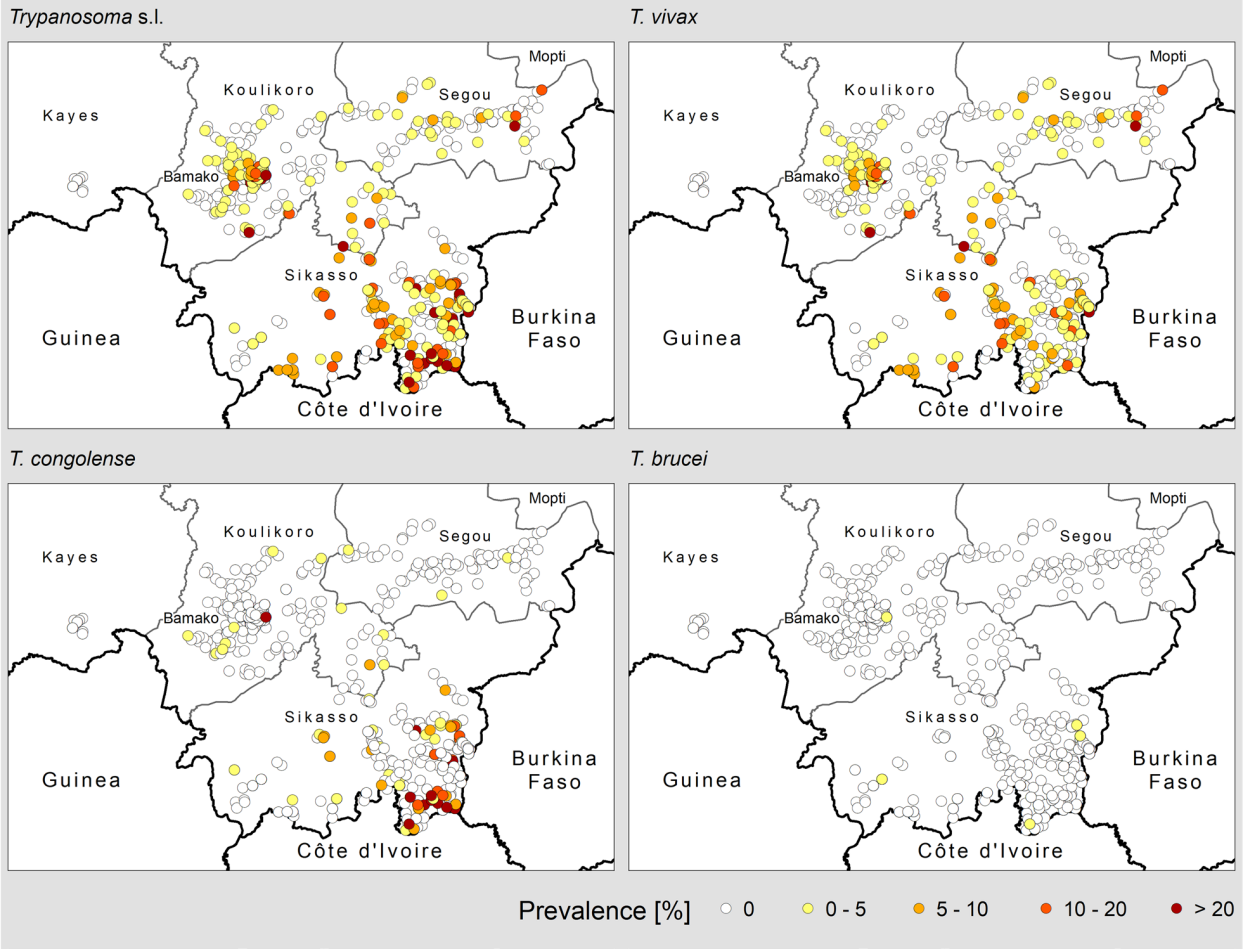
Prevalence of bovine trypanosomosis in Mali. Data collection period: 2000–2018. Diagnostic method: buffy coat technique (BCT) [29]

Hotspots of transmission of bovine trypanosomosis appear to be located in the South (especially, in the areas bordering Côte d’Ivoire and Burkina Faso) and, to a lesser extent, in the South-Centre (in particular, in the peri-urban area of Bamako) (Fig. 3). Average baseline prevalences (i.e. in the absence of coordinated tsetse and trypanosomosis control interventions) ranged between 10% (South), 5% (South-Centre: Koulikoro region), and 1% (South-Centre: Ségou region), for an overall average of 7%.

Looking at the different trypanosome species, overall the average baseline prevalence of *T. congolense* (3.8%) was greater than that of *T. vivax* (2.9%), whilst *T. brucei* prevalence was much lower (0.1%).

With regard to the geographical distribution of AAT, *T. vivax* is fairly widespread and it can be found in all the surveyed areas, including where tsetse densities appear to be very low or tsetse absent (e.g. in the north-eastern part of the Bani river basin, cercles of San and Tominian). In fact, it is known that *T. vivax* circulation can be maintained by the combined effect of mechanical transmission and cattle movement [24]. Compared to *T. vivax*, *T. congolense* seems to have a more focal distribution. The hotspots of *T. congolense* transmission were found in the southern part of the country, with average baseline prevalence in the range of 7%. Despite extensive surveys, very few cases of *T. congolense* have been reported in the South-Centre, with average baseline prevalence for this parasite of approximately 0.5%. Infections with *T. brucei* were rare.

### Database completeness

A high level of completeness was achieved in the database of tsetse and AAT distribution. Regarding the tsetse component, the required information items could be found for the vast majority of the entomological records, in particular geographical coordinates (97%), administrative units [i.e. location name (87%), région (99.9%), cercle (99%) and commune (90%)], date of trapping (83%), duration of trapping (100%), type of trap (100%), tsetse catches (99.9%), tsetse species (99.9%), tsetse apparent densities (99.9%), and possible interventions against tsetse (100%).

Regarding the AAT component, a similarly high level of completeness was achieved for geographical coordinates (99.9%), administrative units [i.e. location name (100%), région (100%), cercle (100%), and commune (99.9%)], survey period (100%), sample size (100%), number and breed of infected animals (100%) and the related prevalence (100%), PCV (86%), sampling strategy (i.e. random or purposeful) (74%), chemotherapy (28%) and possible interventions against tsetse (100%).

## Discussion

The systematic data review for the period 2000–2018 enabled to provide a synoptic, spatially-explicit picture of the available information on tsetse and AAT occurrence in Mali. This picture updates the latest national maps of tsetse distribution, which dated back to 1997 [7], and provides for the first time a national level compilation of AAT occurrence.

However, a number of gaps still affect our knowledge of the distribution of the disease and its vectors in Mali. These gaps are not to be ascribed to the present exercise of data collation, harmonization and geo-referencing, which was highly successful. Instead, they are related to inherent limitations in the existing datasets, and in particular to their geographical and temporal coverage, animal species range, and diagnostic methods.

Regarding the geographical coverage, the main gap affecting data on tsetse distribution is the almost complete lack of information from the western part of the country, as well as the fact that the northern limit of the distribution is not accurately delineated. Other smaller gaps also affect the more intensely investigated South-Centre and South. Furthermore, areas in the northern-eastern and northern part of the country would require entomological surveys to confirm their conjectured tsetse-free status and to ascertain the distribution and epidemiological role of other biting flies (i.e. *Tabanus* spp. and *Stomoxys* spp.).

The geographical coverage of AAT surveys broadly mirrors the one for tsetse, and it is affected by the same gaps. The complete lack of information on AAT occurrence in the northern-eastern and northern part of the country is arguably a more serious issue. This is because, as it is the case in other countries [24], this lack of data may hide a broader AAT distribution than may be inferred from the tsetse distribution alone.

With regard to the temporal coverage, data older than 2000 were not included in the present review for time and resources constraints. However, the main gap in time is arguably related the very limited availability of data for the period 2014–2018. This lack of recent information owes to serious funding issues in the past few years, which brought AAT control and surveillance activities to a virtual stall.

Another limitation in the available epidemiological datasets was the total reliance on the BCT as a diagnostic method, which is known to underestimate the true prevalence of the disease. Furthermore, data on AAT in domestic animals other than cattle are lacking. This is an important limitation in a country where small ruminants are much more numerous than cattle.

With regard to tsetse flies, the results of our mapping for the period 2000–2018 are consistent with observations and trends reported in previous decades [7, 18, 19]. *Glossina palpalis gambiensis* was found in all the surveyed areas in the South, South-Centre and West, and in particular along the main rivers (i.e. Bani and Niger). These data do not show any clear distributional change as compared to older reviews [7], although comparisons are difficult to make because of the differences in the geographical coverage of the surveys and in the mapping methodologies.

As opposed to *G. p. gambiensis*, the distribution of *G. tachinoides* is limited to the southern part of the country. Moreover, there seems to be evidence of a further southwards shift of its northern limit and of a reduction in apparent densities over the past two decades [7].

Regarding the tsetse species of the savannah group historically found in Mali, very few specimens of *G. m. submorsitans* were identified, which confirms the decline documented in past decades [7]. Furthermore, no capture of *G. longipalpis* was reported. These observations are in line with the general trend in other African regions, where the disappearance of savannah species from large areas can be ascribed to the progressive fragmentation and removal of natural vegetation, and it may be exacerbated by climatic stress [7, 31, 32].

With regard to the occurrence of bovine trypanosomosis, the atlas indicates that infections with *T. vivax* are more widely distributed as compared to *T. congolense*. This observation can be explained by the well-known ability of this species to be mechanically transmitted by non-cyclical vectors, and therefore to circulate in areas with low or no tsetse infestation. With regard to the prevalence and distribution of *T. brucei*, it is reported to be very low throughout the study areas. However, as compared with *T. vivax* and *T. congolense*, it is likely that the underestimation of *T. brucei* prevalence be comparatively higher because of the lower level of parasitaemia generated by infections with this species.

## Conclusions

The atlas of tsetse and bovine trypanosomosis confirms that trypanosomosis remains a major animal health problem in Mali and that more efforts should be made for its control, especially in the southern-central and southern regions that show a higher prevalence of AAT. These are the zones of cotton production in Mali where AAT severely constraints the use of draught animals. The atlas is a tool that can be used to target control activities, as well as to plan new surveys to fill the knowledge gaps and update results [24]. Updating is particularly important in order to fill the almost complete lack of data and field activities of the last five years. In this context, carrying out new entomological surveys appears more feasible and affordable, while parasitological surveys are more resource-intensive, and therefore challenging. In terms of geographical gaps, further tsetse and AAT data collection should include the North and North-East where virtually no information is available. In these areas, which are expected to be tsetse-free, the occurrence of trypanosomosis (in particular caused by *T. vivax*) cannot be ruled out because of the combined effect of animal movement and mechanical transmission by non-cyclical vectors. Moreover, it is desirable that the atlas be expanded to include data on livestock species other than cattle. Furthermore, more accurate diagnostics (e.g. molecular tools such as polymerase chain reaction) could be used to increase the sensitivity of detection techniques currently in use. The strong involvement of all stakeholders in providing field data was critical to the successful completion of the atlas. The atlas also contributed to the standardization of recording sheets used in the field to capture data on tsetse and AAT. The initiative benefited from and contributed to building capacity through GIS training-on-the-field workshops for the involved managers and focal points. However, further strengthening the technical capacity of the CCLMT, especially in data management, is imperative for the sustainability of the achievements of the atlas. The atlas is also a fundamental tool for Mali to advance in the PCP for AAT. In fact, the creation of a national level information system on tsetse and AAT is considered as one of the main activities to be conducted in Stage 1, with a view to prioritizing areas and strategies of interventions in subsequent stages. Finally, the methodology developed for the present tsetse and AAT atlas could be applied to other animal diseases, including infections with other trypanosomatids such as *T. evansi* (i.e. surra). A version of this article in French is available in Additional file 3: Text S3.

## Supplementary information

**Additional file 1: Text S1.** List of published sources that contributed to generating distribution maps of tsetse and African animal trypanosomosis in Mali. Reporting period: January 2000 - December 2018. The list contains the 14 sources that have been identified as containing spatially-explicit data on tsetse and animal trypanosomosis in Mali.

**Additional file 2: Text S2.** The structure of the database on tsetse and African animal trypanosomosis in Mali.

**Additional file 3: Text S3.** Version of the article in French.

## Data Availability

Relevant data are within the paper and its Additional files. The bulk of the data on the tsetse and AAT occurrence in Mali is the property of the Government of Mali, Direction Nationale des Services Vétérinaires, Cellule de Coordination de la Lutte contre les Mouches tsé-tsé et les Trypanosomoses animales (CCLMT), and data can be requested to: Direction Nationale des Services Vétérinaires, PO Box: 220, Bamako, Mali, Phone: +223 20 226193/222023, Website: http://mep.gouv.ml/.

## Abbreviations

AAT: African animal trypanosomosis
AfDB: African Development Bank
BCT: buffy coat technique
CCLMT: Cellule de Coordination de la Lutte contre les Mouches tsé-tsé et les Trypanosomoses animales (Coordination Unit for Tsetse and Animal Trypanosomosis Control)
CNRA: Comité National de la Recherche Agronomique (National Committee for Agronomic Research)
FAO: Food and Agriculture Organization of the United Nations
GPS: Global Positioning System
HAT: human African trypanosomosis
IAEA: International Atomic Energy Agency
ITC: insecticide-treated cattle
ITT: insecticide-treated targets
LCV: Laboratoire Central Vétérinaire (Central Veterinary Laboratory)
NEPAD: New Partnership for Africaʼs Development
NGA: National Geospatial-Intelligence Agency
PAAT: Programme against African trypanosomosis
PATTEC: Pan-African Tsetse and Trypanosomosis Eradication Campaign
PCP: progressive control pathway
PCV: packed cell volume
PLMT: Projet de Lutte contre les Mouches tsé-tsé et les Trypanosomoses animales au Mali (Tsetse Fly and Animal Trypanosomosis Control Project in Mali)
UCLT: Unité Centrale de Lutte contre les tsé-tsé et les Trypanosomoses (Central Unit for Tsetse and Trypanosomosis Control)
UTM: Universal Transverse Mercator
WGS84: World Geodetic System 1984

## References

[1] Swallow BM (2000). Impacts of trypanosomiasis on African agriculture.

[2] Alsan M (2014). The effect of the tsetse fly on African development. Am Econ Rev..

[3] Giordani F, Morrison LJ, Rowan TG, de Koning HP, Barrett MP (2016). The animal trypanosomiases and their chemotherapy: a review. Parasitology..

[4] Taylor K, Authié EML, Maudlin I, Holmes PH, Miles MA (2004). Pathogenesis of animal trypanosomiasis. The trypanosomiases.

[5] Desquesnes M, Dia ML (2004). Mechanical transmission of *Trypanosoma vivax* in cattle by the African tabanid *Atylotus fuscipes*. Vet Parasitol..

[6] Büscher P, Cecchi G, Jamonneau V, Priotto G (2017). Human African trypanosomiasis. Lancet..

[7] Djiteye A, Moloo SK, Foua BIK, Touré M, Boiré S, Bengaly S (1997). Réactualisation des données sur la répartition des glossines au Mali. Rev Elev Med Vét Pays Trop..

[8] Cecchi G, Mattioli RC, Cecchi G, Mattioli RC (2009). Global geospatial datasets for African trypanosomiasis management: a review. Geospatial datasets and analyses for an environmental approach to African trypanosomiasis.

[9] Bass B, Bagayoko M, Traore D, Kone F (2014). Prospections des glossines et autres mouches piqueuses dans les cercles de Sikasso et Kadiolo au Mali en prélude à une campagne de suppression. Bull Anim Health Prod Afr..

[10] Franco JR, Cecchi G, Priotto G, Paone M, Diarra A, Grout L (2018). Monitoring the elimination of human African trypanosomiasis: update to 2016. PLoS Neglect Trop Dis..

[11] WHO (2013). Control and surveillance of human African trypanosomiasis.

[12] Kabayo JP (2002). Aiming to eliminate tsetse from Africa. Trends Parasitol..

[13] Diall O, Cecchi G, Wanda G, Argiles-Herrero R, Vreysen MJB, Cattoli G (2017). Developing a progressive control pathway for African animal trypanosomosis. Trends Parasitol..

[14] Sumption K, Domenech J, Ferrari G (2012). Progressive control of FMD on a global scale. Vet Rec..

[15] FAO/OIE. Global Strategy for the Control and Eradication of PPR. 2015.

[16] El Idrissi A (2012). A stepwise approach for progressive control of brucellosis in animals and humans. EMPRES Transbound Anim Dis Bull..

[17] FAO (2012). Developing a stepwise approach for rabies prevention and control.

[18] Ashton DR, Goodwin JT, Ba A, Cisse A. Répartition des mouches tsé-tsé en République du Mali. Texas: Texas Agricultural Experiment Station (TAMU); 1979: 47–54.

[19] Ford J, Katondo KM (1977). Maps of tsetse flies (*Glossina*) distribution in Africa, 1973 according to sub-generic groups on scale of 1:5 000 000. Bull Anim Health Prod Afr..

[20] Mattioli RC, Cecchi G, Paone M, Argilés Herrero R, Simarro PP, Priotto G (2016). The programme against African trypanosomosis. EMPRES Anim Health.

[21] Hursey BS (2001). The programme against African trypanosomiasis: aims, objectives and achievements. Trends Parasitol..

[22] Cecchi G, Paone M, Argiles Herrero R, Vreysen MJ, Mattioli RC (2015). Developing a continental atlas of the distribution and trypanosomal infection of tsetse flies (*Glossina* species). Parasit Vectors..

[23] Cecchi G, Paone M, Feldmann U, Vreysen MJB, Diall O, Mattioli RC (2014). Assembling a geospatial database of tsetse-transmitted animal trypanosomosis for Africa. Parasit Vectors..

[24] Ahmed SK, Rahman AH, Hassan MA, Salih SEM, Paone M, Cecchi G (2016). An atlas of tsetse and bovine trypanosomosis in Sudan. Parasit Vectors..

[25] Leak SGE, Ejigu D, Vreysen MJB (2008). Collection of entomological baseline data for tsetse area-wide integrated pest management programmes.

[26] Mungube EO, Diall O, Baumann MP, Hoppenheit A, Hinney B, Bauer B (2012). Best-bet integrated strategies for containing drug-resistant trypanosomes in cattle. Parasit Vectors..

[27] Mungube EO, Vitouley HS, Allegye-Cudjoe E, Diall O, Boucoum Z, Diarra B (2012). Detection of multiple drug-resistant *Trypanosoma congolense* populations in village cattle of south-east Mali. Parasit Vectors..

[28] Challier A, Laveissiere C (1973). A new trap for catching *Glossina*: description and field trials. Cahiers ORSTOM Sér Entom Méd Parasitol..

[29] Murray M, Murray PK, McIntyre WIM (1977). An improved parasitological technique for the diagnosis of African trypanosomiasis. Trans R Soc Trop Med H..

[30] National Geospatial-Intelligence Agency: NGA GEOnet Names Server (GNS). 2019.

[31] Courtin F, Rayaissé J-B, Tamboura I, Serdébéogo O, Koudougou Z, Solano P (2010). Updating the northern tsetse limit in Burkina Faso (1949–2009): impact of global change. Int J Env Res Pub He..

[32] Dao B, Hendrickx G, Sidibé I, Belem AMG, De La Rocque S (2008). Impact de la sécheresse et de la dégradation des aires protégées sur la répartition des trypanosomoses bovines et de leurs vecteurs dans le bassin versant de lʼOti au nord du Togo. Rev Elev Méd Vét Pays Trop..

