# Supplementary material for: A national atlas of tsetse and African animal trypanosomosis in Mali
Source: Parasit Vectors. 2019 Oct 9;12:466. doi: 10.1186/s13071-019-3721-3 (PMC6784336; doi:10.1186/s13071-019-3721-3)
Supplement: Supplementary file 1 — Additional file 1: Text S1. List of published sources that contributed to generating distribution maps of tsetse and African animal trypanosomosis in Mali. Reporting period: January 2000 - December 2018. The list contains the 14 sources that have been identified as containing spatially-explicit data on tsetse and animal trypanosomosis in Mali. [file 13071_2019_3721_MOESM1_ESM.docx]

# Additional file 1: Text S1

# List of published sources that contributed to generating distribution maps of tsetse and African animal trypanosomosis in Mali

Reporting period: January 2000 – December 2018. The list contains the 14 sources that have been identified as containing spatially-explicit data on tsetse and animal trypanosomosis in Mali.

1. Vreysen MJB, Balenghien T, Saleh KM, Maiga S, Koudougou Z, Cecchi G, et al. Release-recapture studies confirm dispersal of *Glossina palpalis gambiensis* between river basins in Mali. PLoS Neglect Trop D. 2013;7:e2022

2. Hoppenheit A, Bauer B, Steuber S, Terhalle W, Diall O, Zessin KH, et al. Multiple host feeding in *Glossina palpalis gambiensis* and *Glossina tachinoides* in southeast Mali. Med Vet Entomol. 2013;27 2:222-5.

3. Mungube EO, Vitouley HS, Allegye-Cudjoe E, Diall O, Boucoum Z, Diarra B, et al. Detection of multiple drug-resistant *Trypanosoma congolense* populations in village cattle of south-east Mali. Parasite Vector. 2012;5 1:155.

4. Vitouley HS, Mungube EO, Allegye-Cudjoe E, Diall O, Bocoum Z, Diarra B, et al. Improved PCR-RFLP for the detection of diminazene resistance in *Trypanosoma congolense* under field conditions using filter papers for sample storage. PLoS Neglect Trop D. 2011;5 7:e1223.

5. Hoppenheit A, Steuber S, Bauer B, Ouma EM, Diall O, Zessin K-H, et al. Host preference of tsetse: an important tool to appraise the Nagana risk of cattle in the cotton zone of Mali. Wien klin Wochenschr. 2010;122 3:81–6.

6. Grace D, Randolph T, Diall O, Clausen P-H. Training farmers in rational drug-use improves their management of cattle trypanosomosis: A cluster-randomised trial in south Mali. Prev Vet Med. 2008;83 1:83–97.

7. Mungube EO, Diall O, Baumann MP, Hoppenheit A, Hinney B, Bauer B, et al. Best-bet integrated strategies for containing drug-resistant trypanosomes in cattle. Parasite Vector. 2012;5:164

8. Clausen PH, Bauer B, Zessin KH, Diall O, Bocoum Z, Sidibe I, et al. Preventing and containing trypanocide resistance in the cotton zone of West Africa. Transbound Emerg D. 2010;57 1‐2:28–32.

9. Marquez JG, Vreysen MJB, Robinson AS, Bado S, Krafsur ES. Mitochondrial diversity analysis of *Glossina palpalis gambiensis* from Mali and Senegal. Med Vet Entomol. 2004;18 3:288–95.

10. Bass B, Bagayoko M, Traore D, Kone F. Prospections des glossines et autres mouches piqueuses dans les cercles de Sikasso et Kadiolo au Mali en prélude à une campagne de suppression. Bull Anim Health Prod Afr. 2014;62 3:213–24.

11. Bocoum Z, Diarra M, Maiga HM, Sanogo M, Sylla MSM, Diall O. Prevalence in the Bovine Trypanosomiasis Kadiolo Circle. J Community Med Health Edu. 2012;2 9.

12. Bass B, Diall YG, Boire S, Diarra A, Fofana AA, Samake T. Prevalence of bovine trypanosomiasis in Kadiolo and Sikasso in Mali before the startup of a control programme. Bull Anim Health Prod Afr. 2014;62 3:225–31.

13. Bass B, Traore D, Maiga BM, Bengaly S, Diakite B, Kone F. Impact du changement global sur la repartition spatiale des glossines dans le cercle de Bougouni au Mali. Rev Malienne Infectiol Microbiol. 2014; 1:28–33.

14. Bocoum Z, Diarra M, Maiga H, Sanogo I, Traoré Y, Traoré O. African Animal Trypanosomiasis (TAA) in the Zone of Project Management Sustainable Livestock Endemic (Progebe) Mali: Results of Entomological and Parasitological Surveys. J Community Med Health Edu. 2012;2 10.
