# Supplementary material for: A national atlas of tsetse and African animal trypanosomosis in Mali
Source: Parasit Vectors. 2019 Oct 9;12:466. doi: 10.1186/s13071-019-3721-3 (PMC6784336; doi:10.1186/s13071-019-3721-3)
Supplement: Supplementary file 2 — Additional file 2: Text S2. The structure of the database on tsetse and African animal trypanosomosis in Mali. [file 13071_2019_3721_MOESM2_ESM.docx]

# Additional file 2: Text S2

# The structure of the database on tsetse and African animal trypanosomosis in Mali

## Tsetse flies

### Table “entomological data”

1. TSETSE_ID. A unique numeric identifier of each record in the Table.
2. SOURCE_ENTOM. Standardized naming for the source containing the data recorded for the corresponding survey. The file name also includes the date and the name of the geographical surveyed area.
3. ADMIaN1 (*région*). Name of the first subnational administrative unit where the site is located.
4. ADMIN2 (*cercle*). Name of the second subnational administrative unit where the site is located.
5. ADMIN3 (*communes*). Name of the third subnational administrative unit where the site is located.
6. LOCATION_NAME. Name of the site where the study was conducted (i.e. usually the closest village to the trapping site).
7. TRAP_NAME. Identification code as recorded in the file from the field.
8. LAT. Latitude of the study site in decimal degrees (Datum: WGS84).
9. LONG. Longitude of the study site in decimal degrees (Datum: WGS84). LAT/LONG coordinates are specific to the site listed in LOCATION_NAME.
10. SOURCE_GEO. Source of geo-positioning (usually the source itself).
11. YEAR. Year of the survey
12. START_DATE_TIME. Starting date (day/month/year) of the survey.
13. END_ DATE_TIME. Ending date (day/month/year) of the survey.
14. TRAP_TIME (*JOURS*). Duration of trapping (in days).
15. TRAP_TYPE (*piège*). Type of trap.
16. TRAP_ATTR (*ODOUR*). Odour attractant used in the traps.
17. G_P_GAMBIENSIS_F. Number of *G. palpalis gambiensis* (sex=female) caught.
18. G_P_GAMBIENSIS_M. Number of *G. palpalis gambiensis* (sex=male) caught.
19. G_P_GAMBIENSIS_T. Number of *G. palpalis gambiensis* (total) caught.
20. G_TACHINOIDES_F. Number of *G. tachinoides* (sex=female) caught.
21. G_TACHINOIDES_M. Number of *G. tachinoides* (sex=male) caught.
22. G_TACHINOIDES_T. Number of *G. tachinoides* (total) caught.
23. GMS_M. Number of *G. morsitans submorsitans* (sex=female) caught.
24. GMS _F. Number of *G. morsitans submorsitans* (sex=male) caught.
25. GMS _T. Number of *G. morsitans submorsitans* (total) caught.
26. GLOSSINE_T. Total number of flies caught.
27. G_P_GAMBIENSIS_F_AD. *G. palpalis gambiensis* (sex=female) apparent density (flies/trap/day).
28. G_P_GAMBIENSIS_M_AD. *G. palpalis gambiensis* (sex=male) apparent density (flies/trap/day).
29. G_P_GAMBIENSIS_T_AD. *G. palpalis gambiensis* (total) apparent density (flies/trap/day).
30. G_TACHINOIDES_F_AD. *G. tachinoides* (sex=female) apparent density (flies/trap/day).
31. G_TACHINOIDES_M_AD. *G. tachinoides* (sex=male) apparent density (flies/trap/day).
32. G_TACHINOIDES_T_AD. *G. tachinoides* (total) apparent density (flies/trap/day).
33. GMS_M_AD. *G. morsitans submorsitans* (sex=female) apparent density (flies/trap/day).
34. GMS_F_AD. *G. morsitans submorsitans* (sex=male) apparent density (flies/trap/day).
35. GMS_T_AD. *G. morsitans submorsitans* (total) apparent density (flies/trap/day).
36. GLOSSINE_T_AD. Flies (total) apparent density (flies/trap/day).
37. TSETSE_INTERVENTIONS: it reports what interventions against tsetse were ongoing in the study area at the time of the survey, or in the recent past prior to the survey.

## African animal trypanosomosis

### Table “Data sources”

1. SOURCE_ID. A unique numeric identifier of the source examined.
2. INITIALS. Initials of the first author of the input file or scientific paper. Where there is more than one initial, they are separated with a space, e.g. V A.
3. AUTHOR. Name of the first author of the input file or scientific paper.
4. YEAR. Year of generation of the input file (or year of publication of the scientific paper).
5. REPORT_TYPE. Type of the report (e.g. excel file, file from the field, mission or technical report, scientific paper, conference proceedings).
6. PUBLISHED. ‘Yes’ identifies scientific papers, whereas ‘No’ refers to any other document.
7. AAT_DATA. ‘Yes’ identifies documents containing spatially referenced data on AAT occurrence and/or prevalence.
8. TSETSE_DATA. ‘Yes’ identifies documents containing spatially referenced data on tsetse absence presence and/or abundance.
9. FILE_NAME. Standardized naming for PDF files, including author(s), year of publication and surveyed area.
10. INSTITUTION. Name of the national institution or project that generate the data source.

### Table “Geographic data”

1. LOCATION_ID. A unique numeric identifier of the site.
2. LOCATION_NAME. Name of the site where the study was conducted (usually the closest village). As a rule, the name as reported in the paper is recorded.
3. ADMIN1. Name of the first subnational administrative unit (*région*) where the site is located (as reported in the input file).
4. ADMIN2. Name of the second subnational administrative unit (*cercle*) where the site is located (as reported in the input file).
5. ADMIN3_PAPER. Name of the third subnational administrative unit (*commune*) where the site is located (as reported in the input file).
6. LAT. Latitude of the study site in decimal degrees (Datum: WGS84).
7. LONG. Longitude of the study site in decimal degrees (Datum: WGS84). LAT/LONG coordinates are specific to the site listed in LOCATION_NAME.
8. GEO_SOURCE. Source of geo-positioning (usually the input file itself, or one of the available gazetteers).

### Table “Epidemiological data”

1. EPI_ID. A unique numeric identifier of the survey. For different reasons, a single study/paper may include a number of separate surveys, which will result in different values for the SURVEY_ID. For example, if AAT is investigated separately for different animal species (e.g. cattle, goats, sheep, etc.), different SURVEY_ID will identify the data for the different species. Also, if more than one diagnostic technique is used in the same study and if this entails different estimates of AAT prevalence, different rows/SURVEY_IDs will be used to record the different estimates.
2. LOCATION_ID. The numeric identifier of the site where the survey was carried out (it is extracted from the corresponding field in the table ‘Geographic data’).
3. SOURCE_ID. A unique numeric identifier of the source examined (it is extracted from the corresponding field in the table ‘Data sources’).
4. MONTH_ST. Starting month of the survey.
5. YEAR_ST. Starting year of the survey.
6. MONTH_EN. Ending month of the survey.
7. YEAR_EN. Ending year of the survey.
8. SAMPLE_SIZE. Number of bovines sampled.
9. SPECIES_AN. Species of animal (i.e. Cattle only).
10. BREED_AN. Animal breed (e.g. *N’dama*, Zebu or *Méré*).
11. AGE_AN. Age of animals.
12. SEX_AN. Sex of animals.
13. HUSB_AN. Animal husbandry system (prevalently sedentary). Attention is given to whether animals are kept in a prevalently sedentary system, or semi-pastoral/transhumant one, or pastoral. More in general, all aspects that have implications in terms of exposure to infection are noted, including grazing versus zero-grazing, extensive versus intensive, commercial versus small-hold enterprises, etc.

*Infections with individual trypanosome species/subspecies/subgroups*

1. Tv. Number of animals positive to the test for *Trypanosoma vivax*.
2. Tc. Number of animals positive to the test for *T. congolense*.
3. Tb. Number of animals positive to the test for *T. brucei s.l.*
4. T. Number of animals positive to the test for any of the three species of trypanosomes under study (*T. vivax*, *T. congolense*, and *T. brucei*).

Note that animals diagnosed with a mixed infection (infections with more than one species/sub-species/subgroup of trypanosomes) are included in the counts for the respective infections with individual species.

1. TPR [%]. Total AAT Prevalence (in percentage), including infections with any of the three species of trypanosomes under study (*T. vivax*, *T. congolense*, and *T. brucei*).

Separate values for the prevalence of individual species of trypanosomes are also included in the database, but they are not listed in this document.

1. AAT_PRESENCE. ‘Yes’ if the African animal trypanosomosis is present, “No” if the disease is not present. This field might be useful when the input file only reported the absence or presence of AAT, but it reported neither the number of infections, not the prevalence.
2. DIAGNOSTIC. Diagnostic method used for trypanosome identification (Buffy coat technique -BCT- only).
3. PCV. Average Packed-Cell-Volume for the survey herd.
4. TSETSE_INTERVENTIONS. It reports what interventions against tsetse were ongoing in the study area at the time of the survey, or in the recent past prior to the survey.
5. CHEMOTHERAPY. It includes information related to the use of therapeutic and/or prophylactic antitrypanosomal drugs.
6. SAMPLING_STRATEGY. It describes whether a random approach was used (e.g. in a study designed to assess the general epidemiological situation in an area), or a purposeful one (e.g. where specific villages/areas/herds/animals are investigated because of some peculiar features of theirs).
7. LONGITUDINAL. It describes whether data were extracted from a longitudinal study.
