# Supplementary material for: A national atlas of tsetse and African animal trypanosomosis in Mali
Source: Parasit Vectors. 2019 Oct 9;12:466. doi: 10.1186/s13071-019-3721-3 (PMC6784336; doi:10.1186/s13071-019-3721-3)
Supplement: Supplementary file 3 — Additional file 3: Text S3. Version of the article in French. [file 13071_2019_3721_MOESM3_ESM.docx]

# Additional file 3: Text S3

# Un atlas national des tsé-tsé et de la trypanosomose animale africaine au Mali

Boucader Diarra^1^, Modibo Diarra^1^, Oumar Diall^2^, Boubacar Bass^3^, Youssouf Sanogo^1^, Etienne Coulibaly^1^, Mahamadou Sylla^3^, Weining Zhao^4^, Massimo Paone^4^ et Giuliano Cecchi^4*^

^1^Direction Nationale des Services Vétérinaires, Cellule de Coordination de la Lutte contre les Mouches tsé-tsé et les Trypanosomoses animales (CCLMT), Bamako, Mali

^2^Ministère de l’Agriculture, Comité National de la Recherche Agronomique (CNRA) Bamako, Mali

^3^Ministère de l’Elevage et de la Pêche, Bamako, Mali

^4^Organisation des Nations Unies pour l’Alimentation et l’Agriculture (FAO), Division de la Production et de la Santé Animales, Rome, Italie

^*^ Auteur Correspondant : [giuliano.cecchi@fao.org](mailto:giuliano.cecchi@fao.org)

Adresses email:

BD: diarrab@gmail.com

MD: [modibodiarra76@gmail.com](mailto:modibodiarra76@gmail.com)

OD: [odiall@afribonemali.net](mailto:odiall@afribonemali.net)

BB: [drbassl@yahoo.fr](mailto:drbassl@yahoo.fr)

YS: [youssoufsanogo22@gmail.com](mailto:youssoufsanogo22@gmail.com)

EC: [eticouli@yahoo.fr](mailto:eticouli@yahoo.fr)

MS: [msylla57@yahoo.fr](mailto:msylla57@yahoo.fr)

WZ: [weining.zhao@fao.org](mailto:weining.zhao@fao.org)

MP: [massimo.paone@fao.org](mailto:massimo.paone@fao.org)

GC: [giuliano.cecchi@fao.org](mailto:giuliano.cecchi@fao.org)

# Résumé

**Contexte** : La trypanosomose transmise par les glossines est une maladie tropicale négligée mortelle et un défi majeur pour l'agriculture mixte (agriculture-élevage) en Afrique subsaharienne. Elle est causée par plusieurs espèces du genre *Trypanosoma*. Des informations sur la présence de glossines et de la trypanosomose animale africaine (TAA) sont disponibles pour différentes régions du Mali. Cependant, ces données n’ont jamais été harmonisées ni centralisées, ce qui empêche l’élaboration de cartes épidémiologiques complètes et limite la planification des actions de contrôle basée sur des preuves. Pour relever ce défi, nous avons créé une base de données géospatiale dynamique sur la distribution des glossines et de la TAA au Mali.

**Méthodes :** Un dépôt numérique contenant les données épidémiologiques collectées entre 2000 et 2018 a été constitué. En plus de publications scientifiques, le dépôt comprend des fiches de données de terrain, des rapports techniques et autre littérature grise. Les données ont été vérifiées, harmonisées, géoréférencées et intégrées dans une seule base de données spatialement explicite.

**Résultats** : Pour la composante « glossines », environ 19 000 enregistrements, correspondant à 6 000 emplacements de piégeage distincts et à 38 000 mouches, ont été inclus dans la base de données. *Glossina palpalis gambiensis* était l'espèce la plus répandue et la plus abondante. Elle a été trouvée dans le sud, le centre-sud et l'ouest du pays. *Glossina tachinoides* n'a été trouvé que dans le sud. Seuls quelques spécimens de *Glossina morsitans submorsitans* ont été détectés. Pour la composante « TAA », environ 1 000 enregistrements d’enquête ont été inclus, ce qui correspond à 450 sites d’enquête distincts et à 37 000 bovins testés. La TAA a été trouvée dans toutes les régions étudiées, bien que les données pour le nord et le nord-est du pays exempts de glossines soient insuffisantes. Les espèces dominantes étaient *Trypanosoma vivax* et *Trypanosoma congolense*, tandis que les infections à *Trypanosoma brucei* étaient beaucoup moins nombreuses.

**Conclusions :** L'atlas des glossines et de la TAA au Mali fournit une vue synoptique de la situation des vecteurs et de la maladie au niveau national. Néanmoins, d'importantes lacunes géographiques affectent le nord, le nord-est et l'ouest, ainsi qu'un manque cruel de données au cours des cinq dernières années. La trypanosomose reste un problème majeur de santé animale au Mali. Cependant, malgré sa prévalence et sa distribution, les activités de surveillance et de contrôle sont actuellement très limitées. Des efforts devraient être faits pour renforcer le contrôle progressif de la TAA au Mali, et l'atlas fournit un nouvel outil d'identification des domaines d'intervention prioritaires.

**Mots clés :** Mali, glossines, trypanosomose animale africaine, SIG, atlas, base de données, épidémiologie.

# Contexte

La trypanosomose animale (également appelée «nagana») transmise par les glossines entrave la production agricole de plus de 10 millions de km^2^ en Afrique subsaharienne, dont certaines représentent le plus grand potentiel de développement de la production agricole du continent [1]. Il a été soutenu, historiquement, que la trypanosomose réduisait la capacité des populations africaines à générer un excédent agricole et que la performance économique actuelle du continent est toujours fortement affectée par la tsé-tsé [2].

La trypanosomose animale africaine (TAA) est une maladie débilitante due à des parasites protozoaires unicellulaires du genre *Trypanosoma* (ordre des Kinetoplastida) [3]. Elle affecte les ruminants, les chameaux, les équidés, les porcs et les carnivores. Ses principaux agents pathogènes sont *T. vivax*, *T. congolense*, *T. brucei* et *T. simiae* [4]. La TAA est transmise de manière cyclique par la piqûre de glossines (*Glossina* spp.) infectées, mais l'infection, en particulier par *T. vivax*, peut également être transmise mécaniquement par d'autres mouches piqueuses (notamment *Tabanus* et *Stomoxys* spp.) [5]. Deux sous-espèces de *T. brucei*, à savoir *T. b. rhodesiense* et *T. b. gambiense*, sont responsables de la forme humaine de la trypanosomose, également appelée maladie du sommeil [6], tandis que *T. b. brucei* n'est pas pathogène pour l'homme.

Le Mali est un pays à vocation agro-pastorale. Cependant, il est incapable de satisfaire ses besoins en produits d'origine animale, notamment la viande, le lait et les produits laitiers, en raison de la très faible productivité. Au niveau national, on estime que jusqu'à 3 millions de bovins sont exposés au risque de trypanosomose [7, 8], qu’elle est la principale maladie à transmission vectorielle du pays. La trypanosomose est particulièrement répandue dans les régions au potentiel agricole le plus élevé (par exemple, pour la production de coton). Selon les dernières estimations, l'infestation par les glossines serait de 240 000 km^2^ [9]. En ce qui concerne la trypanosomose humaine africaine (THA), aucun cas n'a été enregistré au Mali depuis plus de vingt ans [10], le dernier cas ayant été détecté en 1995 [11].

Dans le présent document, nous nous référons à cinq zones géographiques au Mali, à savoir le sud (région de Sikasso), le centre-sud (au sud des régions de Ségou et Koulikoro, cette dernière comprenant la zone périurbaine de la capitale Bamako), la région de l'ouest (partie sud de la région de Kayes), du nord (parties septentrionales des régions de Kayes, Koulikoro et Ségou) et du nord-est (régions de Mopti, Tombouctou, Gao et Kidal).

Au Mali, les glossines ne se rencontrent que dans les zones du sud, du centre-sud et de l'ouest. La dernière étude sur la répartition des glossines au Mali, publiée il y a plus de vingt ans [7], a signalé la présence de quatre espèces. Les plus abondants à savoir *Glossina palpalis gambiensis* et *G. tachinoides* appartenaient tous deux au groupe riverain, tandis que *G. morsitans submorsitans et G. longipalpis* (deux espèces du groupe savane) étaient beaucoup moins répandus. En ce qui concerne les trypanosomes transmis par les glossines infectant les animaux, *T. congolense, T. vivax* et *T. b. brucei* sont les trois principales espèces au Mali.

Les autorités maliennes considèrent que la lutte contre les glossines et la trypanosomose est un élément stratégique important pour accroître la production agricole, assurer la sécurité alimentaire et améliorer la santé humaine et animale. En fait, depuis les années 1970, un certain nombre d'institutions, de projets et de programmes, dont certains axés sur la recherche, se sont concentrés sur la lutte contre la trypanosomose. Il s'agit notamment du Laboratoire Central Vétérinaire (LCV), de l'Unité Centrale de Lutte contre les mouches tsé-tsé et les Trypanosomoses (UCLT) et du Projet de Lutte contre la Mouche tsé-tsé et la Trypanosomose animale au Mali (PLMT). Le Mali a également adhéré à la Campagne Panafricaine d'Eradication des glossines et des trypanosomoses (PATTEC), une initiative de l'Union Africaine approuvée par les chefs d'État et de gouvernement africains [12].

En 2015, la Cellule de Coordination de la Lutte contre les Mouches tsé-tsé et les Trypanosomoses animales (CCLMT) a été créée et chargée de coordonner les interventions contre les glossines et la trypanosomose au niveau national. La CCLMT est appuyée par le LCV, en particulier pour les questions liées à la recherche opérationnelle.

La CCLMT a récemment élaboré une nouvelle stratégie nationale conforme au cheminement de contrôle progressif (PCP) pour la TAA [13]. Les PCP sont des approches progressives basées sur les risques, développées initialement pour la fièvre aphteuse [14], puis adaptées à un certain nombre d'autres maladies [13, 15, 16, 17]. L'une des exigences fondamentales pour progresser dans le PCP est la cartographie du risque de la TAA pour une prise de décision basée sur des preuves. Cela nécessite une bonne connaissance, spatialement explicite, de la TAA et de la présence de glossines. Cependant, comme c'est le cas dans la plupart des pays touchés, aucune carte nationale de la distribution et de l'endémicité de la TAA n'est disponible au Mali, et les cartes de la distribution des glossines les plus utilisées remontent aux années 1970 [18, 19], avec seulement des mises à jour sporadiques au cours des deux décennies suivantes [7]. Le manque d'informations synoptiques sur la maladie et ses principaux vecteurs limite la possibilité de planifier des actions de lutte de manière rationnelle et d'estimer l'impact des interventions. Pour relever ce défi, la CCLMT a lancé en 2015 le développement d'une base de données géo spatiale dynamique sur la TAA et la répartition des glossines au Mali (c'est-à-dire l'Atlas). L'initiative soutenue par l'engagement du gouvernement malien, bénéficie de l’appui technique de la FAO dans le cadre du Programme de Lutte contre la Trypanosomose Africaine (PLTA) [20, 21]. Le développement de cet outil a été accompagné d'un renforcement des capacités en matière de gestion de données et de systèmes d'information géographique (SIG).

# Méthodes

L'Atlas des glossines et la TAA au Mali suit globalement la méthodologie développée par la FAO pour l'Atlas continental [22, 23], qui avait déjà été adaptée au niveau national au Soudan [24]. Cependant, bien que l'atlas continental de la FAO ne repose que sur des publications scientifiques revues par des pairs, les atlas nationaux visent à inclure toutes les données épidémiologiques collectées dans le pays, qu'elles soient publiées ou non.

L'atlas des glossines et de la TAA au Mali comprend des données recueillies sur la période 2000-2018. La majorité des données ont été fournies par des institutions nationales impliquées ou ayant été impliquées dans la recherche et le contrôle des glossines et de la trypanosomose (UCLT, LCV, PLMT et CCLMT, ce dernier jouant également un rôle de coordination dans l’initiative).

## Les sources de données

Les données utilisées pour l’atlas ont normalement été collectées dans le contexte des activités de lutte contre la TAA. Elles comprennent à la fois des enquêtes de référence (avant l’intervention) et des enquêtes de surveillance (pendant ou après l’intervention). Les données collectées dans ces enquêtes sont rarement publiées. Une source de données supplémentaire est fournie par les activités de recherche, dont les résultats sont généralement diffusés par le biais de publications scientifiques. La liste complète des articles scientifiques dont les résultats ont été inclus dans l’atlas est fournie dans le Fichier supplémentaire 1 : Texte S1. Pour ces publications, les données brutes non publiées ont été obtenues des auteurs et utilisées pour la réalisation de l’atlas national (à la différence de l’atlas continental de la FAO, où les informations et les données pouvaient être extraites directement des publications).

Du point de vue géographique, le centre et le sud du pays ont été les principales cibles des activités de contrôle et de surveillance au cours des deux dernières décennies et ont donc fourni une quantité substantielle de données pour l’atlas. Un projet réalisé entre 2006 et 2013 dans le cadre de l'initiative PATTEC et financé par la Banque africaine de développement (BAD) constituait une autre source de données importante. Le projet ciblait le centre-sud, en particulier le bassin du fleuve Bani et une partie du bassin du Niger (l'accent étant mis sur les zones périurbaines de Bamako).

### Mouches tsé-tsé

Les données entomologiques sur la distribution et l'abondance des glossines sont généralement collectées pour évaluer l'impact des interventions de contrôle. Au Mali, les traitements insecticides des bovins (ITC) et des leurres / pièges (ITT) sont les techniques de lutte anti vectorielle les plus courantes. Les études de base mesurent la présence de vecteurs avant les interventions tandis que les études de suivi évaluent l’impact pendant et après les opérations.

Sur le terrain, les données entomologiques sont généralement enregistrées au moyen de formulaires papier normalisés (feuilles d'enregistrement) [25]. Entre autres éléments, ces formulaires incluent des informations telles que le nom du lieu enquêté, ses coordonnées et ses unités administratives (appelées au Mali régions, cercles et communes), la date de l'enquête, l'heure et la durée du déploiement/retrait des pièges ainsi que le nombre, l'espèce et le sexe des glossines piégées. En moyenne, une seule feuille d'enregistrement peut prendre les données pour 55 pièges environ.

Ces données sont complétées par des informations sur les activités de lutte anti glossinienne menées dans la région, le cas échéant. Ce type d’information est normalement disponible dans les rapports de mission narratifs plutôt que dans les feuilles d’enregistrement des données.

Trypanosomose animale africaine

A l’instar des données sur les glossines, les données sur la TAA sont principalement collectées au cours d'enquêtes épidémiologiques de référence ou de surveillance. Dans ces contextes, les animaux testés sont normalement sélectionnés au hasard et, dans une large mesure, les sites de l'enquête sont également choisis au hasard. En revanche, dans quelques études portant sur la résistance aux médicaments trypanocides, les villages à haut risque où la prévalence et le risque de maladie sont les plus élevés sont choisis à dessein [26, 27].

En termes d’enregistrement des données, les fiches parasitologiques standard incluent les sources de données (y compris l’institution responsable), le nom, les coordonnées géographiques et les unités administratives appartenant au village ou au site d’enquête, la date de l’enquête et la taille de l’échantillon (c.-à-d. le nombre d'animaux testés). Les feuilles comprennent également le nombre d'animaux positifs à la TAA par espèce de trypanosome et l'hématocrite (Rapport du volume des hématies au volume sanguin). Une seule feuille d'enregistrement TAA comprend généralement des informations sur 50 à 100 animaux. L’utilisation éventuelle de médicaments trypanocides (y compris le type de médicament) et l’application éventuelle de traitements épi cutanés du bétail pour la lutte antiglossinienne (c.-à-d. ITC) sont également consignées.

## Structure de l'atlas

L'atlas des glossines et de la TAA au Mali est composé de deux éléments principaux: le dépôt de données et la base de données.

### Dépôt de données

Le dépôt de données comprend des copies numériques de tous les fichiers d'entrée utilisés pour construire la base de données. Il contient des feuilles de calcul, des articles scientifiques (comme indiqué dans le Fichier supplémentaire 1 : Texte S1), des rapports, des mémoires, des thèses et d'autres ouvrages de littérature grise. Au premier niveau du dépôt, les glossines et la TAA sont séparées, puis structurées en sous-dossiers liés aux différentes institutions contributrices. Les noms de fichiers incluent l’heure et la zone des enquêtes afin de faciliter la consultation. La plupart des feuilles d'enregistrement de données de terrain sont stockées dans le dépôt sous forme de feuilles de calcul numériques, car les copies numérisées des copies originales en papier sont rarement disponibles.

### La base de données: composante tsé-tsé

Dans la base de données tsé-tsé, toutes les données sont enregistrées dans un seul tableau (voir Fichier supplémentaire 2 : Texte S2). Pour chaque enregistrement, le tableau inclut la source de données, la localisation (village, par exemple) et les unités administratives associées, les coordonnées géographiques du site de piégeage (latitude et longitude en degrés décimaux sur le datum WGS84), la période de relevé, le type de piège, l'attractif utilisé (le cas échéant) et la durée du piégeage. Les résultats de l’enquête sont enregistrés en termes d’espèces de glossines, de nombre de mouches capturées, de densité apparente (c.-à-d. glossines / piège / jour) et de sexe. Des informations sur la présence éventuelle d'activités de contrôle des glossines dans la zone étudiée sont également enregistrées.

Le piège biconique [28] sans attractifs était le piège utilisé dans toutes les enquêtes. De plus, dans les zones étudiées, les pièges étaient normalement déployés dans l'habitat le plus favorable aux glossines, en particulier dans la végétation riveraine. Les pièges étaient géo référencés avec le GPS et la durée de piégeage était normalement de 24 heures. Dans de rares occasions, des pièges ont été déployés pendant 48 ou 72 heures.

### La base de données: composante trypanosomose animale africaine

Contrairement à la composante tsé-tsé dont la base de données n'inclue qu'une table, les données de la composante TAA sont divisées en trois tables différentes: sources de données, données géographiques et données épidémiologiques. Cette structure à trois niveaux suit plus étroitement celle de l'Atlas continental [23].

La table des sources de données résume les informations sur les fichiers d'entrée stockés dans le dépôt. Un identifiant unique est attribué à chaque source et les informations suivantes sont enregistrées: l'auteur et l'institution ayant généré la source, son titre et l'année de production.

La table des données géographiques comprend des informations sur les sites de l’enquête, à savoir le nom de la localisation, ses coordonnées géographiques et ses unités administratives. Un identifiant numérique unique est attribué à chaque site, utilisé pour lier les données géographiques aux données épidémiologiques. Tous les sites d’enquête sont représentés sous forme d’entités ponctuelles sur les cartes produites.

La table des données épidémiologiques résume les résultats des enquêtes épidémiologiques. Dans cette table, chaque enregistrement comprend: la période d’enquête, la méthode de diagnostic, la taille de l’échantillon (c’est-à-dire le nombre d’animaux testés), les espèces animales, la race animale, la tranche d’âge, le sexe, le système d’élevage et l’hématocrite / PCV au niveau troupeau. Les infections trypanosomiennes sont enregistrées en termes de présence / absence, du nombre d'animaux infectés et du taux de prévalence. Les espèces de trypanosomes (à savoir, *T. vivax*, *T. congolense* et *T. brucei*) sont également répertoriées, ainsi que des infections mixtes de plus d’une espèce. Des informations sur les interventions récentes ou en cours contre les glossines et sur l'utilisation éventuelle de médicaments trypanocides (le cas échéant) sont également consignées. Le type d'échantillonnage d'animaux (aléatoire ou raisonné) est enregistré, si les informations sont disponibles. Les identifiants uniques permettent de relier les enregistrements épidémiologiques aux sources et entités géographiques correspondantes (voir fiche complémentaire 2).

Toutes les données parasitologiques incluses dans l’atlas du Mali concernent une seule espèce domestique (les bovins) et toutes les enquêtes reposaient sur la même méthode de diagnostic, c’est-à-dire la technique du buffy coat (BCT, [29]).

## Processus de développement de l'atlas

Différentes étapes ont été nécessaires pour développer l'atlas. D'abord, toutes les données d'entrée disponibles ont été rassemblées auprès des différentes institutions. Les données disponibles seulement en copie papier, ont été préalablement numérisées (c’est-à-dire entrées dans des feuilles de calcul numériques). Tous les fichiers numériques ont ensuite été rassemblés dans le dépôt de données.

La fusion des différents ensembles de données dans une base de données unique a nécessité un processus systématique de normalisation, d’harmonisation et de vérification. Par exemple, le format des coordonnées géographiques a été normalisé en latitude et longitude (degrés décimaux sur le datum WGS84); de même, les coordonnées projetées (Universal Transverse Mercator (UTM)) enregistrées dans les fichiers d'entrée, ont été converties en latitude et en longitude. Une harmonisation des informations sur les races, les systèmes d’élevage, les noms de lieux géographiques et les unités administratives correspondantes a été également nécessaire pour leur enregistrement.

Un nombre non négligeable d'enregistrements épidémiologiques de la TAA manquaient initialement de coordonnées géographiques basées sur le GPS, même s'ils incluaient normalement le nom du site de l'enquête. Dans ces cas, les coordonnées ont été extraites de sources alternatives, par ex. les répertoires géographiques (les index géographiques contenant les noms et les coordonnées d'une série de localisations géographiques), et en particulier le serveur de noms Geonet développé par la National Geospatial-Intelligence Agency (NGA) des États-Unis [30].

De plus, les fichiers d’entrée manquaient souvent d’informations sur les interventions contre les glossines ou l’utilisation des médicaments trypanocides dans les zones d’étude au moment des enquêtes. En effet, les feuilles d'enregistrement de données standard ne prévoyaient pas de colonne pour enregistrer ce type d'informations. En conséquence, des efforts supplémentaires étaient nécessaires pour tenter en partie de combler ces lacunes et d’enregistrer les informations correspondantes dans la base de données.

# Résultats

Environ 1 024 feuilles d’enregistrement de données ont été incluses dans le dépôt de l’atlas (soit 305 pour la glossine et 719 pour la composante TAA, respectivement). L'examen de la littérature publiée et de la littérature grise a également permis d'identifier 23 documents éligibles. Ces documents comprennent quatorze articles publiés dans des revues scientifiques (Fichier supplémentaire 1 : Texte S1), huit documents supplémentaires, dont cinq thèses de doctorat, une maîtrise et trois thèses de Licence. Les résultats de la cartographie des glossines et de la TAA sont résumés à la figure 1.

## Distribution des glossines

En ce qui concerne la composante tsé-tsé de la base de données, environ 6 000 sites de piégeage ont été enregistrés, ce qui correspond à 19 000 enregistrements entomologiques. Les coordonnées géographiques ont pu être identifiées pour environ 90% des sites de piégeage. Environ 38 000 captures de glossines ont été incluses dans la base de données.

Ces enquêtes entomologiques ont principalement concerné le centre-sud et, dans une moindre mesure, le sud (région de Sikasso). Les données sont très limitées dans l'ouest (région de Kayes) et totalement absentes dans le nord et le nord-est.

Les glossines étaient présentes pratiquement dans toutes les zones étudiées (Figure 2), à l’exception de la partie nord-est du bassin de la rivière Bani. La grande majorité des glossines répertoriées dans l’atlas (98%) appartiennent à l’espèce *G. palpalis gambiensis*, les 2% restants appartenant à *G. tachinoïdes*. Seuls quatre *G. morsitans submorsitans* ont été trouvés. Du point de vue géographique, *G. p. gambiensis* s'est avéré être plus répandu dans les zones étudiées, tandis que la présence de *G. tachinoïdes* s'est limitée au sud du pays (région de Sikasso).

## Distribution de la trypanosomose animale africaine

Le statut d'infection trypanosomienne de 36 728 bovins testés a été enregistré dans la composante TAA de la base de données, ce qui correspond à 465 sites d'enquête distincts.

La TAA a été trouvée dans toutes les régions étudiées infestées par les glossines, bien que des informations limitées soient disponibles dans l'ouest (région de Kayes). Aucune information n'est disponible pour les vastes zones exemptes de glossines dans le nord et le nord-est du pays.

Les foyers de transmission de la trypanosomose bovine semblent être situés au sud (en particulier dans les régions limitrophes de la Côte d'Ivoire et du Burkina Faso) et, dans une moindre mesure, au centre-sud (en particulier dans la zone périurbaine de Bamako) (Figure 3). Les prévalences moyennes dans les études de base (c.-à-d. en l’absence d’interventions coordonnées de lutte contre les glossines et la trypanosomose) étaient comprises entre 10% (sud), 5% (région centre-sud - Koulikoro) et 1% (région centre-sud - Ségou) pour une moyenne globale de 7%.

En ce qui concerne les différentes espèces de trypanosomes, la prévalence moyenne initiale de *T. congolense* (3,8%) était globalement supérieure à celle de *T. vivax* (2,9%), tandis que celle de *T. brucei* était beaucoup plus faible (0,1%).

Concernant la répartition géographique de la TAA, *T. vivax* est assez répandu et on peut le trouver dans toutes les zones étudiées, y compris celles où les densités de glossines semblent très faibles ou nulles (par exemple dans la partie nord-est du bassin de la rivière Bani, cercles de San et Tominian). En fait, il est connu que la circulation de *T. vivax* peut être maintenue par l'effet combiné de la transmission mécanique et du mouvement du bétail [24]. Comparé à *T. vivax*, *T. congolense* semble avoir une distribution plus focale. Les plus grands foyers de transmission de *T. congolense* ont été trouvés dans le sud du pays, avec une prévalence moyenne de base de l'ordre de 7%. Malgré des enquêtes approfondies, très peu de cas de *T. congolense* ont été rapportés dans le centre-sud, avec une prévalence moyenne initiale d'environ 0,5%. Les infections à *T. brucei* étaient rares et espacées.

## Exhaustivité de la base de données

Un haut niveau d’exhaustivité a été atteint dans la base de données sur la distribution des glossines et de la TAA. En ce qui concerne la composante tsé-tsé, les informations requises ont pu être trouvées pour la grande majorité des enregistrements, en particulier les coordonnées géographiques (97%), les unités administratives [nom de site (87%), région (99,9%), cercle (99 %), commune (90%)], date de piégeage (83%), durée du piégeage (100%), type de piège (100%), captures de glossines (99,9%), espèces de glossines (99,9%), glossines densités apparentes (99,9%) et interventions possibles contre les glossines (100%).

En ce qui concerne la composante TAA, un niveau de réalisation tout aussi élevé a été atteint pour les coordonnées géographiques (99,9%), les unités administratives [c'est-à-dire le nom du site (100%), la région (100%), le cercle (100%) et la commune (99,9%)], période d'enquête (100%), taille de l'échantillon (100%), nombre et race d'animaux infectés (100%) et prévalence associée (100%), PCV (86%), stratégie d'échantillonnage (aléatoire ou raisonnée (74 %)), les traitements (28%) et les interventions contre les glossines (100%).

# Discussion

L'examen systématique des données de la période 2000-2018 a permis de brosser un tableau synoptique et spatialement explicite des informations disponibles sur la présence des glossines et de la TAA au Mali. Cette image met à jour les dernières cartes nationales de répartition des glossines datant de 1997 [7] et fournit pour la première fois une compilation au niveau national de la présence de la TAA.

Cependant, un certain nombre de lacunes continuent d’affecter nos connaissances sur la répartition de la maladie et ses vecteurs au Mali. Ces lacunes ne doivent pas être attribuées aux pratiques actuelles de collecte, d'harmonisation et de géo référencement des données, qui ont été couronné de succès. Elles sont plutôt liées aux limites inhérentes aux ensembles de données existants, et en particulier à leur couverture géographique et temporelle, à la gamme des espèces animales et aux méthodes de diagnostic.

En ce qui concerne la couverture géographique, la principale lacune affectant les données sur la distribution des glossines est le manque presque complet d'informations provenant de la partie occidentale du pays, ainsi que le fait que la limite nord de la distribution n'est pas fixée avec précision. D'autres lacunes plus petites affectent également les zones sud-centre et sud plus intensément étudiées. En outre, les zones des régions nord-est et nord du pays nécessiteraient des enquêtes entomologiques pour confirmer qu’elles sont indemnes de glossines et pour déterminer la répartition et le rôle épidémiologique d'autres mouches piqueuses (à savoir, *Tabanus* et *Stomoxys* spp.).

La couverture géographique des enquêtes TAA reflète largement celle des glossines et elle est affectée par les mêmes lacunes. L'absence totale d'informations sur la présence de la TAA dans le nord-est et le nord du pays est sans doute un problème plus grave. En effet, comme c'est le cas dans d'autres pays [24], ce manque de données peut masquer une distribution plus large de la TAA que celle pouvant être déduite de la seule distribution des glossines.

En ce qui concerne la couverture temporelle, les données antérieures à 2000 n’ont pas été incluses dans le présent examen à cause des contraintes de temps et de ressources. Cependant, l'écart principal dans le temps est sans doute lié à la disponibilité très limitée de données pour la période 2014-2018. Ce manque d'informations récentes est dû aux problèmes de financement au cours des dernières années, qui ont amené les activités de contrôle et de surveillance de la TAA à un stand virtuel.

Une autre limite des ensembles de données épidémiologiques disponibles était la dépendance totale à la BCT en tant que méthode de diagnostic, connue pour sous-estimer la prévalence réelle de la maladie. De plus, les données sur la TAA chez les animaux domestiques autres que les bovins font défaut. Ceci est une limitation importante dans un pays où les petits ruminants sont beaucoup plus nombreux que les bovins.

En ce qui concerne les glossines, les résultats de notre cartographie pour la période 2000-2018 sont cohérents avec les observations et les tendances rapportées au cours des décennies précédentes [7, 18, 19]. *G. p. gambiensis* a été trouvé dans toutes les zones étudiées du sud, du centre-sud et de l'ouest, et en particulier le long des principaux fleuves (Bani et Niger). Ces données ne montrent pas de changement net de répartition par rapport aux revues plus anciennes [7], bien qu'il soit difficile de faire des comparaisons en raison des différences de couverture géographique des enquêtes et des méthodologies de cartographie.

Contrairement à *G. p. gambiensis*, la répartition de *G. tachinoïdes* est limitée au sud du pays. De plus, il semble y avoir des preuves d'un déplacement supplémentaire de sa limite nord vers le sud et d'une réduction des densités apparentes au cours des deux dernières décennies [7].

En ce qui concerne les espèces de glossines du groupe de savanes historiquement présentes au Mali, très peu de spécimens de *G. m. submorsitans* ont été identifiés, ce qui confirme le déclin documenté au cours des dernières décennies [7]. De plus, aucune capture de *G. longipalpis* n'a été signalée. Ces observations sont conformes à la tendance générale observée dans d'autres régions africaines, où la disparition d'espèces de savane de vastes zones peut être attribuée à la fragmentation et à l'enlèvement progressifs de la végétation naturelle et peut être exacerbée par le stress climatique [7, 31, 32.].

En ce qui concerne la présence de la trypanosomose bovine, l'atlas indique que les infections à *T. vivax* sont plus largement répandues que celles de *T. congolense*. Cette observation peut être expliquée par la capacité bien connue de cette espèce à être transmise mécaniquement par des vecteurs non cycliques, et donc à circuler dans les zones faiblement infestées de glossines. En ce qui concerne la prévalence et la distribution de *T. brucei*, il serait très faible dans toutes les zones de l’étude. Toutefois, en comparaison avec *T. vivax* et *T. congolense*, il est probable que la sous-estimation de la prévalence de *T. brucei* soit comparativement plus élevée en raison du niveau plus bas de parasitémie généré par des infections chez cette espèce.

# Conclusion

L'atlas des glossines et de la trypanosomose bovine confirme que la trypanosomose reste un problème de santé animale majeur au Mali et que des efforts supplémentaires doivent être déployés pour lutter contre cette maladie, en particulier dans les régions du centre-sud et du sud où la prévalence de la TAA est plus élevée. Ce sont les zones de production cotonnière au Mali où la TAA limite fortement l’utilisation des animaux de trait. L'atlas est un outil qui peut être utilisé pour cibler les activités de contrôle, ainsi que pour planifier de nouvelles enquêtes afin de combler les lacunes dans les connaissances et d'actualiser les résultats [24]. La mise à jour est particulièrement importante pour combler le manque presque complet de données et d'activités sur le terrain des cinq dernières années. Dans ce contexte, la réalisation de nouvelles enquêtes entomologiques semble plus faisable et plus abordable, alors que les enquêtes parasitologiques nécessitent davantage de ressources et sont donc difficiles. En termes de lacunes géographiques, la collecte de données supplémentaires sur les glossines et la TAA devrait inclure le nord et le nord-est où pratiquement aucune information n'est disponible. Dans ces zones, qui devraient être exemptes de glossines, la trypanosomose (en particulier causée par *T. vivax*) ne peut pas être exclue en raison de l'effet combiné du mouvement des animaux et de la transmission mécanique par des vecteurs non cycliques. De plus, il est souhaitable que l'atlas soit élargi pour inclure des données sur les espèces d'animaux d'élevage autres que le bétail. En outre, des diagnostics plus précis (par exemple, des outils moléculaires tels que la réaction en chaîne par polymérase) pourraient être utilisés pour augmenter la sensibilité des techniques de détection actuellement utilisées. La forte implication de toutes les parties prenantes dans la fourniture des données de terrain était essentielle à la réussite de l'atlas. L'atlas a également contribué à la normalisation des feuilles d'enregistrement utilisées sur le terrain pour relever les données sur les glossines et la TAA. L'initiative a bénéficié et contribué à renforcer les capacités par le biais d'ateliers de formation aux SIG sur le terrain pour les gestionnaires et les points focaux concernés. Cependant, il est impératif de renforcer encore les capacités techniques de la CCLMT, notamment en matière de gestion des données, pour assurer la pérennité des réalisations de l'atlas. L'atlas est également un outil fondamental pour le Mali pour progresser dans le PCP de la TAA. En fait, la création d'un système d'information au niveau national sur les glossines et la TAA est considérée comme l'une des principales activités à mener au cours de la première étape, en vue de hiérarchiser les domaines et les stratégies d'intervention au cours des étapes suivantes. Enfin, la méthodologie mise au point par le présent atlas des glossines et de la TAA pourrait être appliquée à d’autres maladies animales, y compris les infections par d’autres trypanosomatidés tels que *T. evansi* (c’est-à-dire le surra).

# Fichiers supplémentaires

**Fichier supplémentaire 1 : Texte S1.** Liste des sources publiées ayant contribué à la création de cartes de répartition des glossines et de la trypanosomose animale africaine au Mali.

Description: Période de référence: janvier 2000 - décembre 2018. La liste contient les 14 sources identifiées comme contenant des données spatialement explicites sur les glossines et la trypanosomose animale au Mali.

**Fichier supplémentaire 2 : Texte S2.** Structure de la base de données sur les glossines et la trypanosomose animale africaine au Mali

# References

1. Swallow BM. Impacts of trypanosomiasis on African agriculture. Rome: Food and Agriculture Organization of the United Nations (FAO); 2000.

2. Alsan M. The effect of the tsetse fly on African development. Am Econ Rev. 2014;105:382–410.

3. Giordani F, Morrison LJ, Rowan TG, de Koning HP, Barrett MP. The animal trypanosomiases and their chemotherapy: a review. Parasitology. 2016;143:1862–89.

4. Taylor K, Authié EML. Pathogenesis of animal trypanosomiasis. In: Maudlin I, Holmes PH, Miles MA, editors. The trypanosomiases. Wallingford: CABI Publishing; 2004. p. 331–53.

5. Desquesnes M, Dia ML. Mechanical transmission of *Trypanosoma vivax* in cattle by the African tabanid *Atylotus fuscipes*. Vet Parasitol. 2004;119:9–19.

6. Büscher P, Cecchi G, Jamonneau V, Priotto G. Human African trypanosomiasis. Lancet. 2017;390:2397–409.

7. Djiteye A, Moloo SK, Foua BIK, Touré M, Boiré S, Bengaly S, et al. Réactualisation des données sur la répartition des glossines au Mali. Rev Elev Med Vét Pays Trop. 1997;50:126–32.

8. Cecchi G, Mattioli RC. Global geospatial datasets for African trypanosomiasis management: a review. In: Cecchi G, Mattioli RC, editors. Geospatial datasets and analyses for an environmental approach to African trypanosomiasis. Rome: Food and Agriculture Organization of the United Nations; 2009. p. 1–39.

9. Bass B, Bagayoko M, Traore D, Kone F. Prospections des glossines et autres mouches piqueuses dans les cercles de Sikasso et Kadiolo au Mali en prélude à une campagne de suppression. Bull Anim Health Prod Afr. 2014;62:213–24.

10. Franco JR, Cecchi G, Priotto G, Paone M, Diarra A, Grout L, et al. Monitoring the elimination of human African trypanosomiasis: Update to 2016. PLoS Neglect Trop Dis. 2018;12:e0006890.

11. WHO: Control and surveillance of human African trypanosomiasis. Geneva: World Health Organization; 2013.

12. Kabayo JP. Aiming to eliminate tsetse from Africa. Trends Parasitol. 2002;18:473–5.

13. Diall O, Cecchi G, Wanda G, Argiles-Herrero R, Vreysen MJB, Cattoli G, et al. Developing a progressive control pathway for African animal trypanosomosis. Trends Parasitol. 2017;33:499–509.

14. Sumption K, Domenech J, Ferrari G. Progressive control of FMD on a global scale. Vet Rec. 2012;170:637.

15. FAO/OIE. Global Strategy for the Control and Eradication of PPR. 2015.

16. El Idrissi A. A stepwise approach for progressive control of brucellosis in animals and humans. EMPRES Transbound Anim Dis Bull. 2012;41:4–8.

17. FAO. Developing a stepwise approach for rabies prevention and control. Rome: FAO; 2012.

18. Ashton DR, Goodwin JT, Ba A, Cisse A. Répartition des mouches tsé-tsé en République du Mali. Texas, USA: Texas Agricultural Experiment Station (TAMU); 1979: 47–54.

19. Ford J, Katondo KM. Maps of tsetse flies (*Glossina*) distribution in Africa, 1973 according to sub-generic groups on scale of 1:5 000 000. Bull Anim Health Prod Afr. 1977;25:188–94.

20. Mattioli RC, Cecchi G, Paone M, Argilés Herrero R, Simarro PP, Priotto G, et al. The programme against African trypanosomosis. EMPRES Anim Health 360. 2016;46:9–13.

21. Hursey BS. The programme against African trypanosomiasis: aims, objectives and achievements. Trends Parasitol. 2001;17:2–3.

22. Cecchi G, Paone M, Argiles Herrero R, Vreysen MJ, Mattioli RC. Developing a continental atlas of the distribution and trypanosomal infection of tsetse flies (*Glossina* species). Parasit Vectors. 2015;8:284.

23. Cecchi G, Paone M, Feldmann U, Vreysen MJB, Diall O, Mattioli RC. Assembling a geospatial database of tsetse-transmitted animal trypanosomosis for Africa. Parasit Vectors. 2014;7:39.

24. Ahmed SK, Rahman AH, Hassan MA, Salih SEM, Paone M, Cecchi G. An atlas of tsetse and bovine trypanosomosis in Sudan. Parasit Vectors. 2016;9:194.

25. Leak SGE, Ejigu D, Vreysen MJB. Collection of entomological baseline data for tsetse area-wide integrated pest management programmes. Rome: Food and Agriculture Organization of the United Nations; 2008.

26. Mungube EO, Diall O, Baumann MP, Hoppenheit A, Hinney B, Bauer B, et al. Best-bet integrated strategies for containing drug-resistant trypanosomes in cattle. Parasit Vectors. 2012;5:164.

27. Mungube EO, Vitouley HS, Allegye-Cudjoe E, Diall O, Boucoum Z, Diarra B, et al. Detection of multiple drug-resistant *Trypanosoma congolense* populations in village cattle of south-east Mali. Parasit Vectors. 2012;5:155.

28. Challier A, Laveissiere C. A new trap for catching *Glossina*: description and field trials. Cahiers ORSTOM, Sér Entom Méd Parasitol. 1973;11:251–62.

29. Murray M, Murray PK, McIntyre WIM. An improved parasitological technique for the diagnosis of African trypanosomiasis. TRoy SocTrop Med H. 1977;71:325–6.

30. National Geospatial-Intelligence Agency: NGA GEOnet Names Server (GNS). 2019.

31. Courtin F, Rayaissé J-B, Tamboura I, Serdébéogo O, Koudougou Z, Solano P, et al. Updating the northern tsetse limit in Burkina Faso (1949–2009): impact of global change. Int J Env Res Pub He. 2010;7:1708–19.

32. Dao B, Hendrickx G, Sidibé I, Belem AMG, De La Rocque S. Impact de la sécheresse et de la dégradation des aires protégées sur la répartition des trypanosomoses bovines et de leurs vecteurs dans le bassin versant de lʼOti au nord du Togo. Rev Elev Méd Vét Pays Trop. 2008;61:153–60.
